# Supplementary material for: Complexome profiling of the Chlamydomonas psb28 mutant reveals TEF5 as an early PSII assembly factor
Source: Plant Cell. 2025 Mar 17;37(6):koaf055. doi: 10.1093/plcell/koaf055 (PMC12164590; doi:10.1093/plcell/koaf055)
Supplement: koaf055_Supplementary_Data [file koaf055_supplementary_data.zip › psb28_interactive.zip!/plots/intensity/qualityPlots/peptides/_Cre17.g700750.t1.2.html]

### Cre17.g700750.t1.2

Trivial name:   
SeqCoverage [median]: 20.20  
Mass: 24.35 kDa  
  
 

| Sequence | MissedCleavage | Mass [Da] | wtIntensity | muIntensity | Score |
| --- | --- | --- | --- | --- | --- |
| AQLVAMAPIKDEWVR | 1 | 1725.92 | 0.00 | 18477.64 | 70.53 |
| SPVAEPFAMDVESQSSDGAK | 0 | 2050.92 | 5209.92 | 29595.18 | 138.66 |
|  |  |  | 5209.9 | 48072.8 |  |
